# Attentive immobility: Investigating the emotional-cognitive mechanism underlying conspiracy mentality and Covid-19 preventive behaviors

**DOI:** 10.1371/journal.pone.0294681

**Published:** 2023-11-16

**Authors:** Shuguang Zhao, Jue Zhou, Ting Wang

**Affiliations:** 1 Research Center of Journalism and Social Development, Renmin University of China, Beijing, China; 2 School of Journalism and Communication, Renmin University of China, Beijing, China; 3 New Era International Communication Research Institute, Renmin University of China, Beijing, China; Yeditepe University, TURKEY

## Abstract

While conspiracy theories have received extensive attention in the realm of misinformation, there has been limited research exploring the impact of conspiracy mentality on individuals’ preventive behaviors during acute public health crises. This study investigates how conspiracy mentality may affect compliance with preventive health measures necessary to fight the COVID-19 pandemic, and the underlying emotional and cognitive mediators. Data was collected through a survey among 1878 Chinese respondents at the conclusion of the pandemic. The results indicate that individuals with higher levels of conspiracy mentality are significantly less engaged in preventive behaviors. Furthermore, this correlation is mediated by a sequence of mediating factors, starting from anger leading to institutional distrust and fear leading to perceived risk. Conspiracists’ response mode can be described as a state of "attentive immobility," in which the impact of heightened institutional distrust outweighs their perceptions of risk, ultimately reducing engagement in preventive behaviors during crises. These findings underscore the importance of debunking initiatives that aim to address and mitigate the negative consequences of conspiracy mentality by targeting the mediating psychological processes during future pandemic threats.

## Introduction

On May 5, 2023, the World Health Organization (WHO) declared the end of the COVID-19 "public health emergency of international concern" [1]. First sounded on January 30, 2020, this alert persisted for over three years, during which the world witnessed more than 770 million confirmed cases of the virus [2]. As the disease spreads rapidly worldwide, a plethora of viral misinformation, especially conspiracy theories, has emerged and gained significant traction on social media platforms [3–9]. These propagated conspiracy theories have been recognized as potential impediments to government efforts aimed at implementing preventive health measures to curb the virus’s spread, thereby exacerbating public health crises [10–12]. In the current research, we explore how conspiracy theories influence individual preventive behaviors during the Covid-19 pandemic. We further analyze the mediating roles of emotions, specifically anger and fear, along with cognitive factors like institutional distrust and perceived risk. These insights are instrumental for strategizing against the dissemination of conspiracy theories in future public crises.

### Psychology of conspiracy mentality

Conspiracy theories offer alternative explanations for significant events, suggesting the presence of covert schemes orchestrated by influential and malevolent factions [13]. These theories frequently challenge conventional narratives, positing that official accounts of events are misleading, concealing hidden truths [14]. COVID-19 fosters the creation of conspiracy theories, as such theories tend to emerge during threatening moments of crisis that breed uncertainty [15]. Some of the most prevalent theories include that 5G technology is responsible for the transmission of the virus, that the virus is a deliberate creation as a biological weapon originating from China, and that Bill Gates is exploiting the pandemic to establish global control through mandatory vaccination and surveillance initiatives. Although various conspiracy theories might seem to be irrelevant in their content, they are rooted in varying degrees of mistrust or antipathy against higher-status groups, officials, or established mainstream accounts.

Belief in conspiracy theories is prevalent among the general public, particularly during societal crisis situations such as a pandemic [10, 16]. A social media analysis of COVID-19-related infodemic around the globe revealed that 7.8% of the 2,311 reports in 25 languages from 87 countries were identified as conspiracy theories [17]. In a recent survey regarding COVID-19 conspiracy beliefs, 35% of the Turkish population strongly agreed that COVID-19 was created in a laboratory as a biological weapon, while 28% strongly agreed that events like COVID-19 were caused by a small group with secret goals and 26% strongly agreed that scientists intentionally deceived the public regarding the virus [18]. Moreover, researchers consistently find that endorsing one particular conspiracy statement increases the likelihood of believing seemly unrelated and even mutually incompatible ones [19–22]. These findings have led scholars to argue that a broader worldview, often referred to as a “conspiracy mentality”, may underpin conspiracy beliefs, and this mentality tends to remain stable over time [8, 23–26]. This general worldview, marked by a perception of the world as rife with conspiracies, can contribute to the acceptance of conspiracy theories pertaining to specific events, such as those related to COVID-19.

Conspiracy mentality has important social and health-related consequences [27] including reduced engagement with mainstream politics, climate change initiatives, vaccination programs, and conventional medical treatments [5]. Furthermore, it has been recognized as a potential hindrance to constructive public responses during the pandemic [10]. Drawing this research together, we hypothesize that conspiracy mentality might negatively impact adherence to COVID-19 mitigation measures, and we focus on the emotional and cognitive mechanisms involved.

### Affective intelligence theory

Considering the world has been repeatedly hit by pandemics such as Spanish flu, SARS, and swine flu, it is evident that negative emotions play a crucial role in shaping individuals’ responses to crises [28]. Affective intelligence theory (AIT) posits that emotions serve as an adaptive system that significantly influences our cognition and subsequent behaviors [29]. Emotions are not merely by-products of cognition but play a pivotal role in shaping it. They guide individuals in decision-making processes by signaling when to rely on established habits or when to seek new information [29–33].

Anger and fear, two crucial negative emotions, are often jointly triggered by different types of social threats, and in turn influence individuals’ preferences and behaviors to cope with these threats [30, 34–36]. For instance, data collected from over 12,000 respondents across six European countries demonstrated that exposure to the threat of the COVID-19 pandemic can evoke both anger and fear [28]. Another survey conducted by Erhardt et al. [34] revealed that fear and anger exert contrasting impacts on trust in the government during COVID-19 crises: fear enhances trust in the government, while anger assigns blame for adverse circumstances to the government. Building on AIT, these two specific emotional appraisals of a threat are neural correlates to different regions of the brain and leads to very different downstream behavioral consequences [37, 38]. Anger regulates behavior aimed at securing familiar goals by relying on preexisting convictions. However, encourages individuals to identify novelty and new information, fostering a learning process regarding the threat that could facilitate innovative behavioral responses [34].

### Negative emotions during the COVID-19 pandemic in China

Negative emotions were widespread in times of Covid-19 [39], especially in China, where the strict "zero-COVID" prevention policy enforced for three-years. China was among the first countries to declare COVID-19 a major public health emergency in early 2020. In response, Chinese authorities swiftly implemented rigorous measures, including strict lockdowns, mass testing, extensive contact tracing, enforced quarantine for confirmed and suspected cases, and travel restrictions. These "zero-COVID" measures were maintained for three years, establishing China as a nation with one of the most enduring COVID-19 prevention strategies.

However, in December 2022, with the emergence of new variants and increased global travel, the Chinese government recognized the need for a more comprehensive approach. Subsequently, China transitioned its prevention policy from strict to lenient quickly, adapting to the changing situation [40, 41]. These policy shifts generated mixed feelings among the general population [42]. With the long-lasting restrictions, many people experienced anger and distrust in the government. They longed for a return to a more normal way of life and the resumption of previously halted activities. On the other hand, fear lingered within the public, as the liberalization of measures raised concerns about the potential for increased infections and health risks.

Moreover, conspiracy mentality is inclined to exacerbate emotional issues, particularly in the context of the COVID-19 pandemic and subsequent lockdowns. The perception of vulnerability and attack associated with conspiracy theories is linked to negative psychological states, including low self-esteem, diminished mental health, feelings of powerlessness, fear, and anger [43]. Studies also demonstrate that conspiracy mentality is a risk factor for an increased likelihood of experiencing anxiety, depressive feelings, and distress during COVID-19 lockdowns, regardless of the region [44–46]. It is evident that conspiracy beliefs not only fail to provide any beneficial consequences but also contribute to the reinforcement of negative experiences, such as anxiety, aversion to uncertainty, and existential threats [9].

### Cognitive characteristics and behavioral consequences of conspiracy mentality

Previous research has identified significant associations between conspiracy mentality and various cognitive consequences, a primary one of which is distrust in institutions. Scholars have characterized conspiracy mentality as mistrust, paranoia, and defiance towards powerful groups, authority figures, and established narratives [24, 26, 47]. This distrust is reported to be linked with distrust in science in general, or susceptibility to deceptive pseudoscience [8, 48], and a reduced likelihood of adhering to government guidelines [43]. A study conducted by Chadwick et al. [49] explored the relationship between conspiracy mentality, social media use, online social endorsement, and their implication for vaccine hesitancy. The findings indicated that people scoring higher in conspiracy mentality show significantly greater intention to use social media to discourage vaccination rather than to encourage it. Nevertheless, the precise relationship among conspiracy mentality, distrust, and preventive health behaviors remains unclear, necessitating further investigation.

Conspiracy mentality is also closely associated with higher perceptions of risk or threats, as individuals often embrace conspiracy theories as a response to stress-provoking events. This inclination is driven by various social psychological motives, including the quest for subjective certainty, control, security, and the preservation of a positive self-image or group identity [13, 15]. Additionally, individuals with an underlying conspiratorial reasoning style tend to be more susceptible to strengthening their belief in conspiracy theories when confronted with external stressors [50]. Empirical evidence consistently supports the association between conspiracy beliefs and feelings of uncertainty [51], a perception of diminished socio-political control [24], heightened perceptions of risk, a sense of being under attack [5, 43, 52], and feelings of threat stemming from societal changes [53].

Aside from cognitive characteristics, conspiracy mentality might further influence individuals’ behaviors, particularly concerning preventive health behaviors. Previous research about HIV, vaccines, and other public health topics has shown that belief in conspiracy theories increases people’s resistance against important public health interventions [3, 8, 54]. Examples include resistance to get vaccinations, rejection of conventional medical treatments, and propensity for unsupported or potentially dangerous treatments based on non-mainstream belief systems [3, 6, 13, 54–56]. The influence of conspiracy mentality on medical behaviors also extends to the context of Covid-19 [57]. Studies focusing on Covid-19 related conspiracy theories have found that higher levels of coronavirus conspiracy thinking are associated with lower observance to official guidelines, reduced willingness to undergo diagnostic or antibody tests, hesitancy towards vaccination, and in some cases, intentional engagement in risky behavior [18, 43, 58].

Although multiple studies indicate that conspiracy mentality leads to fewer protective behaviors faced with diseases, its influence can vary depending on individuals’ specific beliefs. For instance, scholars have differentiated between various conspiracy claims and found that they might lead to different levels of containment behavior during a public health crisis. Individuals who perceive COVID-19 as deliberately created and distributed tend to have a more threatening perception of the pandemic and exhibit more containment behavior. On the other hand, those who consider COVID-19 a hoax perceive the pandemic as less threatening and exhibit less adherence to containment measures [59]. These findings imply that the impact of conspiracy mentality on individuals’ adoption of protective behaviors may be mediated by emotions and cognitive factors, but the underlying mechanisms need to be further explored.

### Overview of the current research

While numerous studies have explored the association between conspiracy mentality and preventive health behaviors during pandemic crisis, limited research has delved into the mediating processes that explain these relationships. To date only a small handful of studies have partly investigated the mediating effects of anger, fear, institutional distrust, and perceived risk [5, 7, 10, 36]. An enlightening study by Karić and Međedović [7] examined the relationships between conspiracy beliefs, political trust and behavioral adherence. They found that reduced political trust acts as the mediating factor between conspiracy beliefs and reduced adherence to containment behaviors. Additionally, researchers have observed that distrust in authorities, feelings of powerlessness, disillusionment and perceived dangers of vaccines mediate the negative relationship between anti-vaccine conspiracy beliefs and vaccination intentions [5]. However, these studies are primarily exploratory and have not thoroughly examined the potential mediating effects of emotional and cognitive factors.

In sum, previous research indicates that conspiracy mentality affects preventive health behaviors through a complex interplay of emotional and cognitive mediating mechanisms. However, this process still requires further investigation and clarification. In the current study, we proposed an integrated emotional–cognitive mechanism to illustrate the impact of conspiracy mentality on preventive behaviors. Data was collected from Chinese citizens through an online retrospective survey during China’s "zero-COVID" policy relaxation (March 30—April 18, 2023). We therefore developed the following hypotheses in our model:

**Hypothesis 1 (H1)**: Individuals scoring higher in conspiracy mentality are expected to demonstrate reduced engagement in preventive behavior during the pandemic.
**Hypothesis 2 (H2)**: The impact of conspiracy mentality on individuals’ adoption of preventive behavior is proposed to be mediated by emotions, specifically anger and fear. These emotions, triggered by external threats and crises, are expected to exert their influence through cognitive factors, including perceived risk and institutional distrust, ultimately affecting individuals’ engagement in preventive behavior.
**Hypothesis 3 (H3)**: The exertion process of anger differs from that of fear. Conspiracy mentality is expected to be a positive predictor of both anger and fear. Anger is anticipated to primarily lead to increased institutional distrust, and reduced protective health behavior. In contrast, fear is expected to heighten perceived risk, leading to greater adherence to preventive behavior.

**Fig 1:**
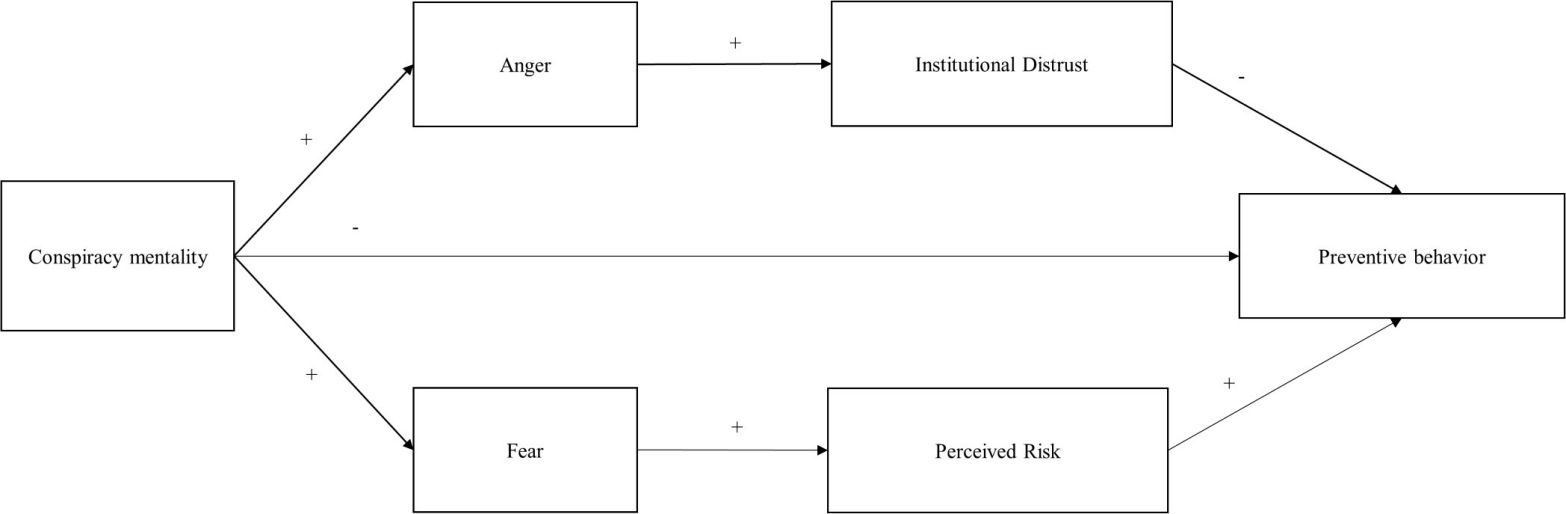
The proposed path model of how conspiracy mentality influences preventive behavior through mediation effects of emotions and cognitive factors.

The structure of the whole model is presented below (Fig 1).

## Method

### Study design and sampling procedure

The data used in this study was collected through an online survey of Chinese residents. The survey was conducted between March 30, 2023, and April 18, 2023, using a Chinese business survey platform (https://survey.work/survey/). Participants were recruited through convenience sampling, and the population consisted of Chinese citizens who were over 18 years old and had internet access. Informed consent was retrieved from participants before they completed the questionnaire.

A total of 2,000 questionnaires were retrieved, and after excluding invalid cases such as incomplete or low-quality replies, or replies completed in unreasonable short time, data of 1878 participants were used for further data analysis. Among the participants, 53.35% were male and 46.65% were female, which is comparable to the gender distribution in the population (51.2% vs. 48.8%). The age distribution of the participants was as follows: 43.13% were between the ages of 18 and 30, 50.47% were between the ages of 30 and 40, 5.32% were between the ages of 40 and 50, and 1.07% were above 50. In terms of education, 66.14% of the participants had a bachelor’s degree or higher, compared to 15.5% in the general population. Regarding residency, 54.74% of the participants lived in big cities, while 45.26% lived in villages or small cities, compared to 27.6% and 72.4% in the population, respectively.

The more detailed socio-demographic breakdown of the sample is presented below in Table 1.

**Table 1:** The demographic distribution of the sample.

Demographic Variables	Year: 2023	
	N	%
**Gender**		
Male	1002	53.35
Female	876	46.65
**Age Group**		
18–23	302	16.08
24–29	508	27.05
30–35	688	36.63
36–40	260	13.84
41–45	78	4.15
46–50	22	1.17
51–60	15	0.8
Over 60	5	0.27
**Highest education**		
Primary school and below	5	0.27
Secondary school	33	1.76
High school	221	11.77
Dazhuan (Junior college)	377	20.07
Undergraduate	1104	58.79
Graduate and upper	138	7.35
**Marital status**		
Single	611	32.53
Married	1252	66.67
Divorced	14	0.75
Widowed	1	0.05
**Living district**		
Village or small cities	850	45.26
Big cities	1028	54.74
**Total**	1878	100.00

### Measures

#### Conspiracy mentality

We adopted items from three established scales measuring conspiracy mentality and selected the statements assessing an individual’s attitude towards generic statements on conspiracy beliefs instead of items mentioning specific historical events or groups. Three scales are adopted and modified as follows. The Generic Conspiracist Beliefs Scale (GCBS) [23] includes 15 items measuring 5 conspiracist facets, which Brotherton et al. identified as government malfeasance, extra-terrestrial cover-up, malevolent global conspiracies, personal well-being and control of information. Each factor was represented by three items. The conspiracy mentality scale [26] contains 12 items tapping into a general propensity to believe in conspiracies. The conspiracy theory ideation scale [60] measures general tendency to believe in any conspiracy with 7 items. After selection, 18 items were included in the questionnaire. Participants were asked to indicate their attitudes towards the statements using a seven-point Likert scale, where 1 represented “strongly disagree” and 7 represented “strongly agree”.

Confirmatory factor analysis showed that two items, namely “I believe the various conspiracy theories on media are totally bullshit (item 5)”, and “there is no reason not to trust the government, intelligence department and media (item 8)”, were reversely related to the construct and were therefore dismissed. The remaining items demonstrated factor loadings above 0.6, indicating their strong association with the construct. After excluding the two reversed items, the scale showed high internal consistency with a Cronbach’s alpha of 0.97.

#### Anger and fear

Anger and fear were assessed using single-item measures. Participants were asked to rate their level of agreement with the statements: “The influences of the Covid-19 pandemic are rising anger/fear in me.” Responses were recorded on a seven-point Likert scale, ranging from 1 (“completely disagree”) to 7 (“completely agree”).

#### Institutional distrust

The institutional distrust of participants was measured with eight items, focusing on how much participants trusted in the mainstream accounts and official news about Covid-19 and the pandemic. Statements were as follows: “I believe the news related to the pandemic is guided/is misleading/is more serious than the facts/is excessively sensationalized/contains elements of exaggeration,” “When I hear news related to the pandemic, I need to spend more time thinking about it/ I need to spend more time thinking about how to minimize the impact of the news.” Participants were asked to rate their agreement with these statements on a seven-point Likert scale, where 1 indicated “completely disagree” and 7 indicated “completely agree”. Higher scores on this scale reflected greater distrust towards the media and news reports related to Covid-19.

In the confirmatory factor analysis, it was found the last two statements had a factor loading lower than 0.6 and was therefore excluded from further analysis. The remaining statements were retained for analysis. The Cronbach’s α is 0.94 for this scale.

#### Perceived risk

Perceived risk was assessed using a scale adapted from the Risk Behavior Diagnosis Scale of Witte [61]. The scale included three items measuring perceived severity. The original general statements related to threats were modified to specifically address the severity of the Covid-19 pandemic. Statements employed to measure perceived severity were as follows: “I believe that the pandemic is severe/serious/significant.” Participants were asked to indicate their level of agreement via a seven-point Likert scale, ranging from 1 (“completely disagree”) to 7 (“completely agree.”) Higher scores on the scale indicated a greater perception of severity and risk associated with the Covid-19 pandemic. The Cronbach’s α is 0.90 for this scale.

#### Preventive behavior

In our assessment of preventive behavior, we included behaviors advised by the World Health Organization to self-protect and prevent the spread of Covid-19 [62]. Behaviors listed in the questionnaire were handwashing, wearing a face mask, covering the mouth and nose while sneezing, and carrying out social distancing. Participants were questioned about how frequently they conducted these behaviors over the past month via a seven-point Likert scale, ranging from 1 (“never”) to 7 (“always”). Higher scores reflecting a greater adherence to health-protective practices. The Cronbach’s α for this scale is 0.82, indicating a good reliability.

## Data analysis

As some measurements presented significant skewness, non-parametric analyses were performed. Table 2 presents the details of mean values, standard deviations, and the Spearman’s rank correlation matrix of the measurement variables. The correlational analysis indicated a significant negative correlation between conspiracy mentality and preventive behavior, providing support for H1. Additionally, anger and institutional distrust displayed significant negative correlation with preventive behavior, and exhibited a positive correlation with each other, partially aligning with the proposed mediational patterns in H2. However, while fear was positively correlated with preventive behavior, the correlation did not reach statistical significance on the 0.01 level.

**Table 2:** Descriptive statistics and Spearman’s rank correlation matrix of the measurement variables.

Variables	Mean	Std. Dev.	(1)	(2)	(3)	(4)	(5)	(6)
(1) Conspiracy mentality	4.01	1.47	1.00					
(2) Fear	4.37	1.85	0.40*	1.00				
(3) Anger	3.98	1.90	0.44*	0.74*	1.00			
(4) Perceived risk	5.23	1.36	0.21*	0.40*	0.31*	1.00		
(5) Institutional distrust	3.48	1.63	0.58*	0.37*	0.45*	0.03	1.00	
(6) Preventive behavior	6.12	0.85	-0.14*	0.01	-0.08*	0.28*	-0.30*	1.00

*p < 0.01.

To investigate the precise mechanisms through which conspiracy mentality influences preventive behavior, we conducted a path analysis utilizing AMOS 24.0 software. The measurement variables, except for fear and anger, were employed as latent constructs in the analysis. Fear and anger, represented as a single factor, were included in the model as observed variables.

### Confirmatory factor analysis

To assess the validity of the measurement model, confirmatory factor analysis (CFA) was conducted before the path analysis, with 6 latent variables constructed by 31 observed indicators. Fear and anger were each represented by a single factor with no unique variables.

Convergent validity was evaluated based on the following criteria: factor loadings, reliability measured by Cronbach’s α, and average variance extracted (AVE). Specifically, two items from the conspiracy mentality scale, two items from the institutional distrust scale, and one item from the preventive behavior scale were excluded from the model since their factor loadings were lower than 0.6. All the factor loadings of the remaining observed indicators were higher than 0.6. All AVE values for the measurements exceeded 0.5. The Cronbach’s α values of all the measurements exceeded 0.8 as displayed above, indicating a good convergent validity. Discriminant validity was evaluated by contrasting the values of AVE with the maximum shared squared variance (MSV). These comparisons revealed that the AVE values for all the measurement variables were greater than their corresponding MSV values, indicating good discriminant validity.

With regards to the overall model fit, results of indexes proved to be satisfactory. Chi-square test: CMIN/DF = 5.476, RMSEA = 0.049 (<0.05), GFI = 0.924 (>0.9), AGFI = 0.908 (>0.9), CFI = 0.962 (>0.95), SRMR = 0.051 (<0.06). We therefore concluded the measurement model is fit for further analysis.

### Path analysis

We constructed the whole structural equation model to test our hypotheses and examine the mediating processes after the validation of the measurement model. Correlational analyses showed significant associations between participant demographics, including age and educational level, with conspiracy mentality, fear and anger. Therefore, age and educational level were incorporated in the path analysis model as control variables. Educational level was quantified in terms of years of education, reflecting the highest level of educational attainment.

The fit indices of the model are as follows. Due to the sensitivity of χ^2^ to sample size, indexes including RMSEA, GFI, AGFI, CFI, and SRMR were also reported. According to Kline [63], if the RMSEA value is lower than 0.05, it can be accepted as a good approximate fit, and the range between 0.05 and 0.08 suggested a reasonable fit. Hu and Bentler [64] noted that a cut of value close to 0.95 for CFI and a cutoff value close to 0.08 for SRMR can be considered as indicators for a satisfactory model fit. According to these criteria, the path analysis proved to be an overall good fit: CMIN/DF = 4.66 (<5), RMSEA = 0.044 (<0.05), GFI = 0.930 (>0.9), AGFI = 0.916 (>0.9), CFI = 0.964 (>0,95), and SRMR = 0.036 (<0.05).

The control variables included in the model were also examined with their relationships with measurement variables. Results showed that educational level had a negative correlation with both anger (β = -0.105, se = 0.020, p < 0.001) and institutional distrust (β = -0.074, se = 0.018, p < 0.001). Age had a positive correlation with perceived risk (β = 0.094, se = 0.022, p < 0.001). Results indicated that people with higher educational attainment were associated with fewer emotions of anger and less institutional distrust, while elderly people experienced higher perceptions of risk faced with the pandemic.

H1 posits that individuals scoring higher in conspiracy mentality are expected to demonstrate reduced engagement in preventive behavior during the pandemic. In support of H1, the total effect of conspiracy mentality on preventive behavior was -0.107 (se = 0.026, p < 0.001, 95% bias-corrected confidence interval = -0.157, -0.055), signifying that conspiracy mentality is associated with significantly less engagement in preventive behavior.

H2 probes into the mediating effects of emotions and cognitive factors in the process by which conspiracy mentality leads to reduced adoption of preventive behavior. To validate the mediation effects, a bootstrap test was conducted using 2,000 bootstrap resamples. Results showed significant indirect effects from conspiracy mentality to preventive behavior (β = -0.110, se = 0.024, p < 0.001, 95% bias-corrected confidence interval = -0.158, -0.062). These findings indicated that the chains of anger leading to institutional distrust and fear leading to perceived risk serve as significant mediators between conspiracy mentality and engagement in preventive behavior, thus supporting H2. Accounting for these significant mediational pathways, the direct effect from conspiracy mentality to preventive behavior was not significant (β = 0.002, se = 0.032, p = 0.898, 95% bias-corrected confidence interval = -0.056, 0.067). This suggests the effect from conspiracy mentality to preventive behavior was fully mediated by the two pathways. Additionally, it is noteworthy that the effect from conspiracy mentality to media trust was partially mediated by anger, with a significant yet relatively small indirect effect of 0.097 (the ratio of indirect effect to the total effect is 16.1%). On the other hand, fear fully mediated the relationship between conspiracy mentality and perceived risk (direct effect = 0.059, se = 0.027, p = 0.021, 95% bias-corrected confidence interval = 0.009, 0.115, which was not significant accounting for the mediation effect). This implies that the effect of conspiracy mentality on perceived risk primarily operates through the path of fear, while increased institutional trust is more directly influenced by conspiracy mentality itself, with only partial mediation by the emotion of anger. The details of the total, direct and indirect effects of the proposed mediational pathways are presented in Table 3.

**Table 3:** Total, direct, and indirect effects of conspiracy mentality and mediators.

	Standardized estimate	SE	CI	*p*-value
**CM to PB**				
Total effect	**-0.107**	**0.026**	**[-0.157, -0.055]**	**0.001**
Direct effect	0.002	0.032	[-0.056,0.067]	0.898
Indirect effect	**-0.110**	**0.024**	**[-0.158, -0.062]**	**0.001**
**Anger to PB**				
Total effect	**-0.154**	**0.036**	**[-0.226, -0.084]**	**0.001**
Direct effect	-0.084	0.037	[-0.157, -0.012]	0.032
Indirect effect	**-0.070**	**0.010**	**[-0.090, -0.051]**	**0.001**
**Fear to PB**				
Total effect	**0.225**	**0.037**	**[0.151,0.298]**	**0.001**
Direct effect	0.079	0.037	[0.007,0.151]	0.031
Indirect effect	**0.146**	**0.016**	**[0.114,0.179]**	**0.001**
**CM to ID**				
Total effect	**0.602**	**0.020**	**[0.561,0.642]**	**0.001**
Direct effect	**0.505**	**0.023**	**[0.459,0.551]**	**0.001**
Indirect effect	**0.097**	**0.011**	**[0.078,0.118]**	**0.001**
**CM to PR**				
Total effect	**0.226**	**0.026**	**[0.173,0.276]**	**0.001**
Direct effect	0.059	0.027	[0.009,0.115]	0.021
Indirect effect	**0.167**	**0.014**	**[0.140,0.195]**	**0.001**

*Note*: Significant effects are bolded for ease of viewing. CM = conspiracy mentality, PB = preventive behavior, ID = institutional distrust, PR = perceived risk. 95% bias-correlated confidence intervals used, with 2,000 bootstrap samples. Controlling for age and education.

H3 explores the divergent effects of anger and fear in the mediational processes. It is hypothesized that conspiracy mentality is simultaneously associated with increased anger and fear. While anger predicts increased institutional distrust, and reduced protective health behavior, fear is expected to be linked with heighten perceived risk, leading to greater adherence to preventive behavior. The results of path analysis supported the hypotheses. Conspiracy mentality was found to be a significant positive predictor for both anger (β = 0.420, se = 0.023, p < 0.001) and fear (β = 0.393, se = 0.024, p < 0.001). Anger was positively correlated with institutional distrust (β = 0.231, se = 0.024, p < 0.001), and institutional distrust was a negative predictor of preventive behavior (β = -0.303, se = 0.032, p < 0.001). On the other hand, fear was found to be a positive predictor of perceived risk (β = 0.425, se = 0.026, p < 0.001), and perceived risk was associated with higher engagement in preventive behavior (β = 0.343, se = 0.030, p < 0.001). To conclude, higher levels of anger among people with elevated conspiracy mentality sparked more distrust in mainstream accounts and official media, ultimately resulting in reduced engagement preventive behavior. Conversely, the heightened fear, driven by conspiracy mentality during times of threat, led to greater perceptions of risk and increased participation in preventive behavior.

**Fig 2:**
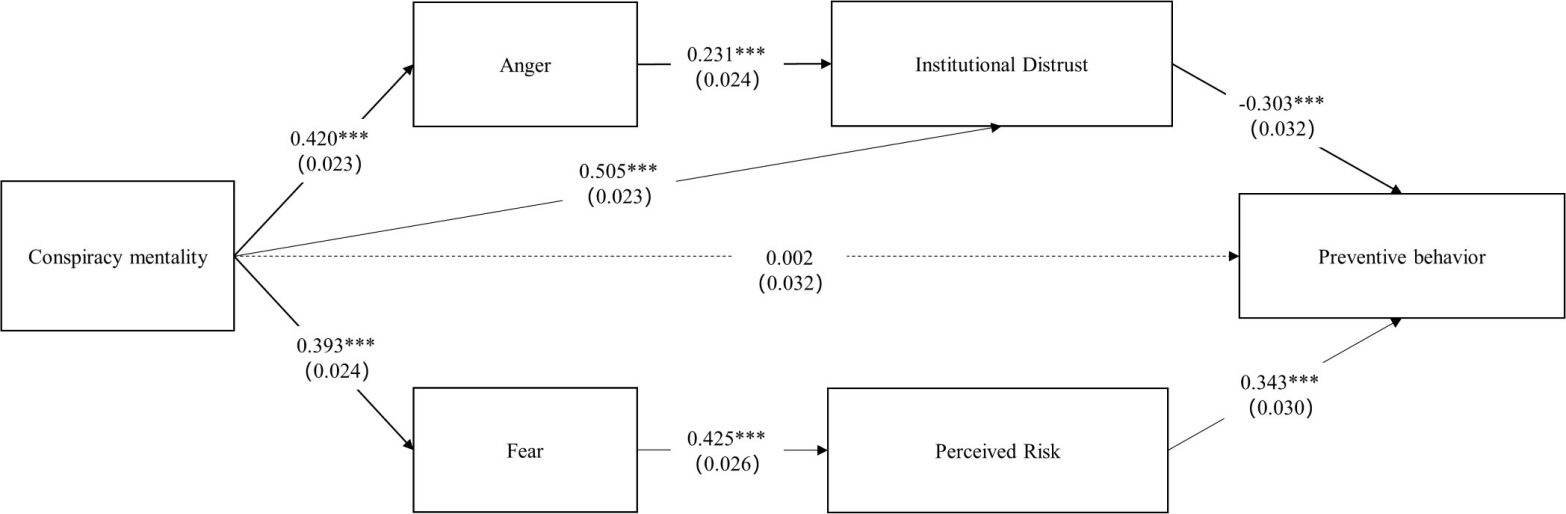
Path model estimation results. Significance: *p < 0.05, **p < 0.01, ***p < 0.001. H1 and H2 tested total and mediation effects, therefore were not directly reflected in the path model. The detailed results of H1 and H2 can be found in Table 3.

Fig 2 presents the revised model with detailed validating results of the hypotheses.

## Discussion

The COVID-19 pandemic has sparked extensive conspiracy theories and instilled strong negative emotions, particularly in the age of the proliferation of social media platforms [65, 66]. The negative consequences of conspiracy mentality are especially concerning during global health emergencies since it may hinder individuals from adhering to government health guidelines [43, 67]. China’s strict three-year "zero-COVID" policy, followed by a dramatic relaxation, provides a valuable case to explore the impact of conspiracy mentality on individuals’ preventive behaviors during a public health crisis, as well as the possible emotional and cognitive mediators involved. We conducted an online survey among Chinese citizens during the relaxation phase of China’s "zero-COVID" policy, which occurred from March 30 to April 18, 2023. The results supported our hypothesis that conspiracy mentality is linked to a reduced adoption of preventive health practices aimed at mitigating the spread of COVID-19. There was also evidence that emotions, including anger and fear, and cognitive factors like institutional distrust and perceived risk, could mediate the relationship between conspiracy mentality and individuals’ coping behaviors.

Firstly, conspiracy mentality has been a prominent feature of the pandemic, resulting in disastrous consequences. It is not an effective approach for dealing with threats and uncertainty; instead, it reinforces negative experiences. In a global health crisis like the COVID-19 pandemic, characterized by rapid spread and high mortality rates, individuals face significant uncertainty and threats. Research has demonstrated that feelings of uncertainty have the potential to foster conspiracy beliefs [68]. In other words, when people perceive a lack of control and order in the world, they may turn to conspiracy beliefs as a means of restoring a sense of order and control. This aligns with the compensatory control theory [69], which suggests that individuals experiencing a loss of personal control seek compensatory mechanisms to restore order, such as believing in a controlling God, a highly competent government, or even embracing abstract beliefs. Despite their commonly malevolent assumptions, conspiracy theories provide a framework for understanding events that threaten the established order [15, 70]. From this perspective, conspiracy mentality could be a compensatory control mechanism. However, contrary to beneficial consequences, our findings indicate that conspiracy mentality might actually exacerbate negative emotions such as anger and fear, thereby hindering the adoption of protective measures during a pandemic. This aligns with the perspective in the existing literature [9] that conspiracy beliefs establish a self-reinforcing cycle characterized by distrust, uncertainty, and conspiratorial thinking.

According to AIT, pandemic threats have the potential to evoke anger and fear, both of which are closely linked to conspiracy mentality. This finding contributes to the body of existing literature, which already identifies conspiracy mentality as a risk factor for experiencing negative emotions during Covid-19 lockdowns [43–46]. Moreover, our findings revealed that individuals with high levels of conspiracy mentality during a health crisis tend to exhibit significantly greater distrust in pandemic-related information and perceive the crisis as more threatening. These results are consistent with the existing literature concerning the cognitive outcomes of conspiracy mentality, encompassing distrust in institutions and mainstream narratives [26, 47], as well as heightened sensitivity to risks or threats during external events [5, 43, 51, 53].

We also expanded our research to explore the mediating process through which conspiracy mentality influences preventive behaviors by way of negative emotions, institutional distrust, and perceived risks. In accordance with AIT, threats generate anger and fear with very different cognitive and behavioral consequences. Fear increases the perception of risk and further encourages a powerful response, but anger removes the trust in institution and reduces the preventive behaviors. More specifically, the mediating effects of these two paths work in contradictory directions: individuals with higher conspiracy mentality experience increased levels of both anger and fear. Greater anger is associated with increased distrust in mainstream accounts and official media, resulting in reduced adoption of protective behaviors. Conversely, heightened fear increases the perception of risk and ultimately results in greater engagement in preventive behaviors. Notably, distrust seems to have a stronger and more stable association with conspiracy mentality as a trait, and it is less influenced by emotions. Additionally, it has a more adverse impact on compliance with preventive measures compared to perceptions of risk.

Furthermore, our results corroborate previous findings indicating that individuals with higher levels of conspiracy mentality are less likely to engage in effective measures to protect themselves from Covid-related risks [10]. This can primarily be attributed to the negative effects of increased anger and institutional distrust, which overshadow the positive influence of fear and perceived risk. These findings provide an explanation for the seemingly paradoxical observation that individuals with higher levels of conspiracy mentality engage in fewer protective behaviors, even though they perceive the crisis as more threatening. This behavioral pattern can be likened to the classic “freeze response” observed in individuals faced with sudden threats or stressful events. In such situations, individuals may be highly alert but find themselves unable to take action or respond effectively to the perceived danger. Some literature has summarized this response as a state of “attentive mobility” [71]. Similar to individuals’ instant responses under pressure, their reaction mode during external crises is determined by their emotions and perceptions of the events. In the case of individuals with conspiracy mentality, their psychological response process should be attributed to the effects of anger and institutional distrust, rather than solely focusing on their superficial perception of higher risks. This finding is consistent with previous literature, which has also demonstrated a link between belief in conspiracy theories and behaviors such as medical inactivity or non-compliance with medical treatments [3, 6, 8, 54, 55].

## Limitations

Several factors need to be considered when interpreting the results of this study.

Firstly, findings of this research are based on a convenience sample limited to Chinese adults with Internet access, which is typically comprised of a younger and more highly educated demographic than the overall population. In an effort to reduce potential biases in our estimates, we included age and educational level as control variables in our model. Previous research has indicated that Covid-19 disproportionately affects the elderly, and individuals aged 60 and above may hold distinct beliefs and behaviors in relation to the pandemic [72, 73]. However, the limited number of respondents aged 60 and above in our sample continues to limit the generalizability of our conclusions primarily to young adults and middle-aged individuals.

Additionally, the use of a self-report methodology to assess emotions and coping behaviors introduced the potential for response biases and common-method variance. These measures might be susceptible to personal interpretation, which could potentially impact the accuracy of the responses. Despite our efforts to ensure the reliability of the measures and conduct data quality checks, the reliance on self-reported data inherently introduced limitations.

Another limitation is the use of a cross-sectional dataset. The data utilized in this study was collected between March and April 2023, which corresponds to the later stages of the Covid-19 crisis in China. During this period, the "zero-covid" policy had been relaxed, and mass Covid-19 testing had ceased. It is important to note that we collected the data just after the peak of widespread contamination across the country [74], indicating that the period can still be considered a crisis for Chinese residents. Unfortunately, despite conducting a similar survey during the initial outbreak of Covid-19, we were unable to obtain a sufficient number of first-wave samples. This limitation prevented us from validating our model using a longitudinal dataset. The cross-sectional design inherently lacks the ability to conclusively establish causality, restricting our capacity to definitively discern the direction of the relationship between conspiracy mentality and preventive behavior. However, given that conspiracy mentality tends to be a relatively stable disposition involving belief in conspiracy theories over time [26], we cautiously identified it as a founding factor leading to emotions, cognitions, and corresponding behaviors. Additionally, we were unable to capture the changes in preventive behavior of individuals with higher levels of conspiracy mentality over time. This limitation hampers our understanding of the long-term and dynamic nature of this relationship.

To address these limitations, future research may benefit from employing complementary study designs. For instance, studies focusing on elderly samples could provide valuable insights given the unique beliefs and behaviors of this demographic in a pandemic. Longitudinal studies or experiments could offer a more comprehensive understanding of the casual relationship between conspiracy mentality and preventive behavior faced with threats and crises.

## Conclusion

This study has provided an exploration of the intricate mechanism by which conspiracy mentality influences individuals’ preventive health behaviors during a public health crisis like the COVID-19 pandemic. Importantly, our findings have shown that fear heightens the perception of risk and motivates a strong self-protective response, whereas anger erodes trust in institutions and reduces compliance.

By revealing the negative correlation between conspiracy mentality and individuals’ engagement in preventive behaviors, along with the mediating pathways involving emotions and cognitive factors, we have developed a preliminary model to clarify this intricate process. The psychological responses of individuals with elevated levels of conspiracy mentality when confronted with environmental risks or threats can be likened to a state of “attentive immobility”, a phenomenon akin to the classic "freeze response" mode [71]. Mediated by the emotion of fear, these individuals experience an increased perception of risk associated with the crisis. Nevertheless, partially mediated by the emotion of anger, they also manifest heightened distrust towards mainstream accounts, experts, and health authorities. The impact of this distrust surpasses the effects of perceived risk, impeding their ability to take effective containment actions. Consequently, individuals with high conspiracy mentality tend to exhibit reduced engagement in preventive behaviors, even though they may possess a heightened awareness of potential risks. These findings underscore the critical importance of comprehending the ramifications of conspiracy theories and the need to implement effective communication strategies aimed at disrupting cognitive and responsive processes.

In conclusion, by applying Affective Intelligence Theory, this study expands our understanding of how conspiracy mentality influences individuals’ preventive behaviors during public health crises and provides valuable insights into its mediating process comprised by emotional and cognitive factors. Our research also provides a valuable case study focused on Chinese examples of Covid-19 conspiracy theories and their psychological implications. This adds a culturally specific dimension to the understanding of the ramification of conspiracy mentality, especially its impact on public health responses. By acknowledging the detrimental impact of conspiracy theories on individuals’ compliance to health guidelines, policymakers, public health professionals, and officials can develop targeted strategies, including debunking projects, to combat conspiracy mentality and foster effective coping modes during future acute health crises.
